# Molecularly Imprinted Polyacrylamide with Fluorescent Nanodiamond for Creatinine Detection

**DOI:** 10.3390/ma12132097

**Published:** 2019-06-29

**Authors:** Reim A. Almotiri, Kathryn J. Ham, Vineeth M. Vijayan, Shane A. Catledge

**Affiliations:** Center for Biophysical Sciences and Engineering (CBSE), Department of Physics, University of Alabama at Birmingham, 421 Campbell Hall, 1300 University Blvd, Birmingham, AL 35294, USA

**Keywords:** nanodiamond, creatinine, fluorescence, molecularly imprinted polymer

## Abstract

Creatinine measurement in blood and urine is an important diagnostic test for assessing kidney health. In this study, a molecularly imprinted polymer was obtained by incorporating fluorescent nanodiamond into a creatinine-imprinted polyacrylamide hydrogel. The quenching of peak nanodiamond fluorescence was significantly higher in the creatinine-imprinted polymer compared to the non-imprinted polymer, indicative of higher creatinine affinity in the imprinted polymer. Fourier transform infrared spectroscopy and microscopic imaging was used to investigate the nature of chemical bonding and distribution of nanodiamonds inside the hydrogel network. Nanodiamonds bind strongly to the hydrogel network, but as aggregates with average particle diameter of 3.4 ± 1.8 µm and 3.1 ± 1.9 µm for the non-imprinted and molecularly imprinted polymer, respectively. Nanodiamond fluorescence from nitrogen-vacancy color centers (NV^−^ and NV^0^) was also used to detect creatinine based on nanodiamond-creatinine surface charge interaction. Results show a 15% decrease of NV^−^/NV^0^ emission ratio for the creatinine-imprinted polymer compared to the non-imprinted polymer, and are explained in terms of changes in the near-surface band structure of diamond with addition of creatinine. With further improvement of sensor design to better disperse nanodiamond within the hydrogel, fluorescent sensing from nitrogen-vacancy centers is expected to yield higher sensitivity with a longer range (Coulombic) interaction to imprinted sites than that for a sensor based on acceptor/donor resonance energy transfer.

## 1. Introduction

The physical, chemical, and biological properties of diamond make it an attractive substitute to organic dyes, fluorescent proteins, and semiconductor nanocrystals (quantum dots) to probe the interaction of biomolecules. In the form of nanodiamonds, these properties provide attractive features that can be used in various biological applications [1,2,3,4,5,6].

Organic dyes and fluorescent proteins have disadvantages such as photo-bleaching which make them unsuitable for prolonged use. An alternative to such dyes and proteins is quantum dots, which are semiconductor nanoparticles with fluorescence capabilities. Quantum dots typically have high tolerance to photo-bleaching, broad excitation ranges, and narrow emission spectra [7]. However, they are often toxic, which may limit their use for testing in humans unless they have undergone surface modification to reduce their toxicity [8]. Nanodiamonds offer a suitable alternative to quantum dots since they not only have high threshold for photo-bleaching but are considered much less toxic than quantum dots [5]. A further advantage of nanodiamonds over quantum dots is that they have more stable photo-physical properties. On the contrary, the surface modifications to quantum dots, either when being functionalized or to reduce toxicity, have been reported to change their photo-physical properties [8]. This limits the extent to which they can be modified and therefore restricts their applications compared to nanodiamonds, which can be extensively surface-modified without losing their photo-physical properties [8,9].

**Figure 1:**
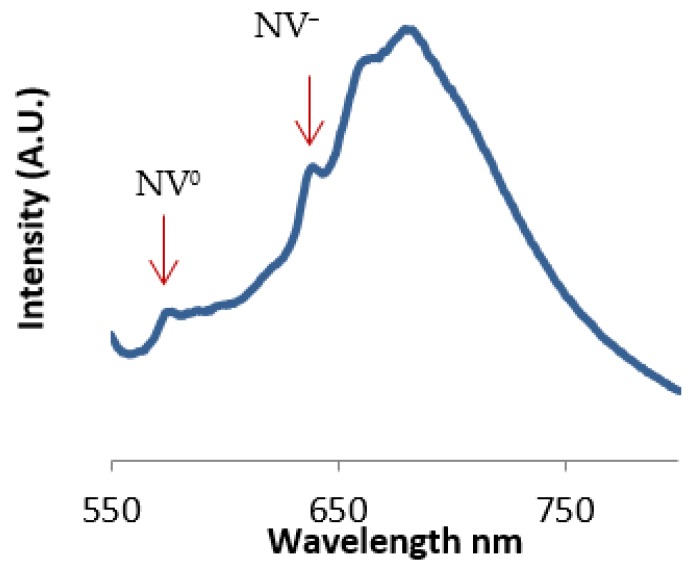
The emission spectrum of nanodiamond and the location of zero phonon lines from both nitrogen-vacancy centers (λ_exc_ = 532 nm).

The nitrogen-vacancy (NV) color center in diamond is a point defect with C3v symmetry in which a nitrogen atom sits next to a vacant position in the lattice [10]. This defect can occur during the synthesis of diamond during chemical vapor deposition or can arise from radiation damage and annealing [11]. The defect exists in two states, the neutral state (NV^0^) and the negatively charged state (NV^−^). The zero-phonon lines for the NV^−^ and NV^0^ states occur at 637 nm and 575 nm, respectively, as shown in Figure 1. For diamonds that contain nitrogen-vacancy color centers, reducing the particle size to the nanometer scale brings these defects close to the surface where they can interact with adsorbates to modify the near-surface band structure [12].

In this work, we show that the fluorescence from nanodiamond, and in particular that from the nitrogen-vacancy zero-phonon lines, is modified when in close proximity to the small molecule creatinine. This is believed to be due to a changing surface chemical potential of the nanodiamond. This is especially the case where nanodiamonds are incorporated into molecularly imprinted polymers (MIP) specifically designed to have an affinity for creatinine in aqueous media, and can therefore be used as a molecular sensor.

The measurement of the amount of creatinine in serum or urine is an important diagnostic test because it is a significant indicator of renal function [13]. Current clinical laboratory assays of creatinine concentrations are based on colorimetry and enzymatic activity [14]. All of these tests are time-consuming, relatively expensive and require complex equipment. While new tests have been developed, they have the same disadvantages as their predecessors: They require equipment and procedures which are more complex and costlier than the molecularly imprinting technique. The current work focuses on the development of a sensitive and specific fluorescence-based assay for the concentration of creatinine in aqueous media using molecularly imprinted polyacrylamide incorporated with nanodiamond as a signal transducer. This may be an improvement over current chemical assays for creatinine because it is faster and cheaper than current methods and can potentially be used as a “Lab-on-a-chip” technology.

Immobilization of carbon dots (CDs) within molecularly imprinted polymers has been shown to be effective for detection of glucose at physiological pH [15]. CDs functionalized with hydroxyl or carbonyl groups formed complexes with a glucose-imprinted polymer through hydrogen bonding. Such hydrogen bonding enabled the CDs to be incorporated into the gel network during the polymerization stage, and thus offered a means for detecting sites of the molecularly imprinted polymers occupied by glucose following subsequent glucose treatment [15]. The fluorescence of the incorporated CDs is determined by the binding or release of glucose from the binding site on the molecularly imprinted polymer. Where glucose binds into its site on the molecularly imprinted polymer, the fluorescence from the CDs is quenched. On the contrary, recovery of the fluorescence is achieved after releasing glucose molecules from a molecularly imprinted polymer-CD network [15].

In this work, we show that fluorescence quenching of nanodiamonds imbedded in a creatinine-imprinted polyacrylamide (PAAm) hydrogel can be used in the detection of this small molecule. This quenching is detectable not only from the overall broad nanodiamond fluorescence (with peak intensity around 700 nm), but also from the intensity ratio of nitrogen-vacancy centers (NV^−^/NV^0^). The ratiometric sensor offers an advantage in that it is specific to surface chemical potentials at the near nanodiamond surface and may be less prone to fluorescence from unwanted background (e.g., polymer-induced fluorescence). The fluorescent sensing is expected to provide reliable sensitivity of trace level quantities of creatinine.

## 2. Materials and Methods

### 2.1. Chemicals

Acrylamide monomer, bis-acrylamide (a cross-linker agent), creatinine powder (99+%), N-hydroxysuccinimide (NHS, 98+%), Tetramethylethylendiamine (TEMED) and ammonium persulfate (APS) were purchased from Fisher Scientific, Waltham, MA, USA. Fluorescent nanodiamond (70 nm average particle size, 1 mg/mL in deionized (DI) water with >300 nitrogen-vacancy centers per particle) was purchased from Sigma-Aldrich (St. Louis, MO, USA).

### 2.2. Preparation of Molecularly Imprinted Polyacrylamide for the Detection of Creatinine

PAAm hydrogels were formed by co-polymerization of acrylamide and bis-acrylamide. The chemical reaction is vinyl addition polymerization initiated by APS and TEMED. TEMED accelerates the rate of formation of free radicals from APS, which, in turn, catalyzes the polymerization process. Polyacrylamide gels are described in terms of two parameters. The total monomer concentration, or %T, is defined as:

%T = (grams acrylamide + grams crosslinker)/total volume (mL) × 100%

The weight percentage of crosslinker, or %C, is defined as:

%C = grams crosslinker/(grams acrylamide + grams crosslinker) × 100%.

Our hydrogel optimization process led to a composition having 15%T and 5%C in 7 mL of DI water. Molecularly imprinted and a non-imprinted polymer (NIP), as control, were prepared under identical conditions to contain 300 µL of 1 mg/mL of nanodiamond suspension in 7 mL of polymerization solution, except that the non-imprinted polymer control was made without addition of creatinine during polymerization. A concentration of 85 µL of 0.3 mM creatinine in 7 mL of molecularly imprinted polymer solution was used in this process. Further details for polymerization, washing and rebinding processes can be found in our previous work [16].

### 2.3. Analysis Method

The photoluminescence of nanodiamond was collected from a modular Raman/fluorescence spectrometer (Dilor, Lille, France) with a 532 nm laser, 1200 groove/mm grating, and a 100× microscope objective. The emission from nanodiamonds was measured before and after the addition of creatinine to the molecularly imprinted and non-imprinted polymers, respectively.

### 2.4. Calculation of Imprinting Factor Based on Quenching of Peak Nanodiamond Emission

The imprinting factor was measured from the change in fluorescence from the peak emission intensity of nanodiamond (around 700 nm) before and after the creatinine quencher was added in the rebinding tests. The Imprinting Factor (IF) is defined as: IF = (Quenching ratio of molecularly imprinted polymer)/(Quenching ratio of non-imprinted polymer), where:

$$Quenching\;ratio\;=\;\frac{\mathrm{Maximum}\;\mathrm{peak}\;\mathrm{intensity}\;\mathrm{of}\;\mathrm{nanodiamonds}\;\mathrm{before}\;\mathrm{the}\;\mathrm{addition}\;\mathrm{of}\;\mathrm{creatinine}\;}{\begin{array}{c} \mathrm{Maximum}\;\mathrm{peak}\;\mathrm{intensity}\;\mathrm{of}\;\mathrm{nanodiamonds}\;\mathrm{after}\;\mathrm{the}\;\mathrm{addition}\;\mathrm{of}\;\mathrm{creatinine} \end{array}}$$

### 2.5. Polymer Synthesis for Stern-Volmer Analysis

PAAm hydrogel was polymerized to the concentration of 15%T and 5%C with 300 μL of 1 mg/mL fluorescent nanodiamond. PAAm samples were prepared with four different creatinine imprinting concentrations below:

Sample 1: Nanodiamond-incorporated molecularly imprinted polymer with 0.82 µM creatinine.
Sample 2: Nanodiamond-incorporated molecularly imprinted polymer with 2.40 µM creatinine.
Sample 3: Nanodiamond-incorporated molecularly imprinted polymer with 4.10 µM creatinine.
Sample 4: Nanodiamond-incorporated molecularly imprinted polymer with 6.20 µM creatinine.

**Figure 2:**
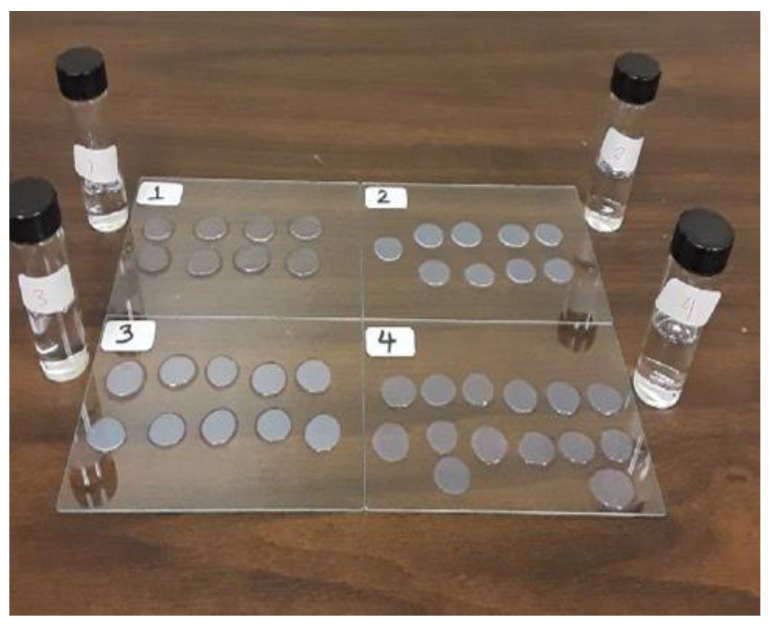
Nanodiamond-incorporated molecularly imprinted polymer hydrogel samples containing the four different concentrations of creatinine (0.82 µM, 2.40 µM, 4.10 µM, 6.20 µM).

After polymerization was completed the hydrogel was washed with DI water to remove the creatinine and other unreacted elements. The washing was repeated 8 times and samples were put in DI water and exposed to ultrasonic vibration for 10 min to help reduce remaining creatinine in the hydrogel. As shown from our previous work, eight washing steps in DI water was effective at removing the majority of creatinine [16]. Several samples from each of the four concentrations were prepared for fluorescence measurements, as shown in Figure 2.

The intensity of the nanodiamond fluorescence (around 700 nm) was measured before and after the addition of creatinine. The Stern-Volmer equation for our experiment is given in general as:

I_0_/I = K[C] + 1

where I_0_ and I are the fluorescence intensity maxima in the absence of creatinine and in the presence of creatinine, respectively; K is the Stern-Volmer constant determined as the slope of the best-fit line to the data; and [C] is the concentration of creatinine.

## 3. Results and Discussion

### 3.1. Fourier Transform Infrared Spectroscopy (FTIR) of Fluorescent Nanodiamond

Nanodiamond surface chemistry can impact relative fluorescence emission from nitrogen-vacancy color centers with different charge states [12]. Therefore, it is important to identify the initial functional groups that exist on the nanodiamond surface in order to establish the charge termination as being predominantly negative or positive. This affects the nanodiamond surface chemical potential for differently charged adsorbates. Such interaction between nanodiamonds and creatinine molecules can occur near and within the binding sites of the molecularly imprinted polymer network [17].

**Figure 3:**
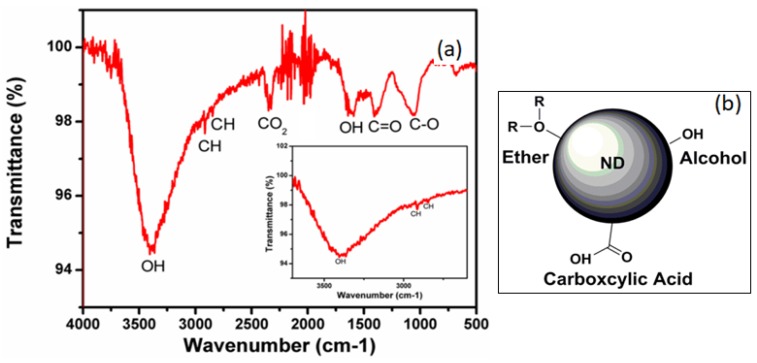
(**a**) Fourier transform infrared spectroscopy (FTIR) spectrum of the as-received fluorescent nanodiamond (inset of the figure represents the spectral region from 2300–3800 cm^−1^ showing OH and CH groups). (**b**) Schematic showing several functional groups that exist on the surface of the as-received nanodiamond.

To explore the nature of surface functionalization of the as-received nanodiamonds, Fourier transform infrared spectroscopy (FTIR) was performed. The FTIR spectrum of the nanodiamonds shown in Figure 3 reveals multiple peaks ranging from 3385 to 1047 cm^−1^. Table 1 describes the complete peak assignment of the FTIR spectral analysis.

The observed peaks were assigned appropriately after referencing with previously reported FTIR spectral interpretation of nanodiamonds [18]. FTIR spectral analysis clearly reveals polyfunctional surface groups such as OH, COOH, ethers and hydrogen. The monomer/crosslinker used in the current study for molecular imprinting process was acrylamide, which was found to undergo hydrogen bonding interactions [19]. We hypothesize that during molecularly imprinted polymer formation, similar hydrogen bonding interactions (both inter- and intra-molecular) occurs between the surface hydroxyl groups of the nanodiamonds with the different hydroxyl groups present on the acrylamide. This could facilitate strong interaction of the nanodiamonds with the polymer network and is expected to result in their stability during washing/rebinding steps. We find that the nanodiamond fluorescence emission lasts after washing the hydrogel (including in an ultrasonic DI water bath). The nanodiamonds used in this work have functional groups with predominantly negative charge termination as shown in the schematic of Figure 3.

### 3.2. Nanodiamond Cluster Size and Fluorescence inside PAAm Hydrogel

**Figure 4:**
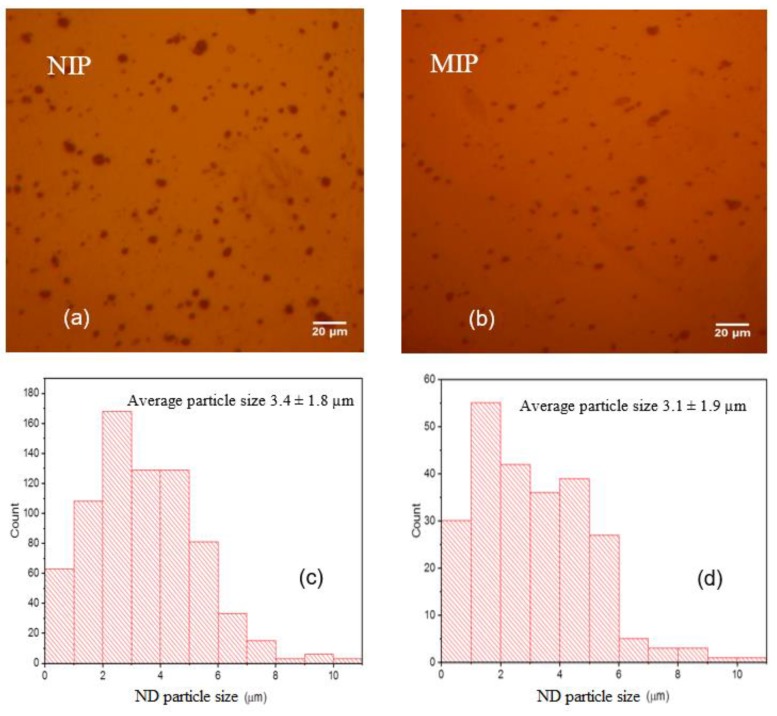
Optical micrographs (**a**,**b**) and particle size distributions (**c**,**d**) for the non-imprinted and molecularly imprinted polymers, respectively.

**Figure 5:**
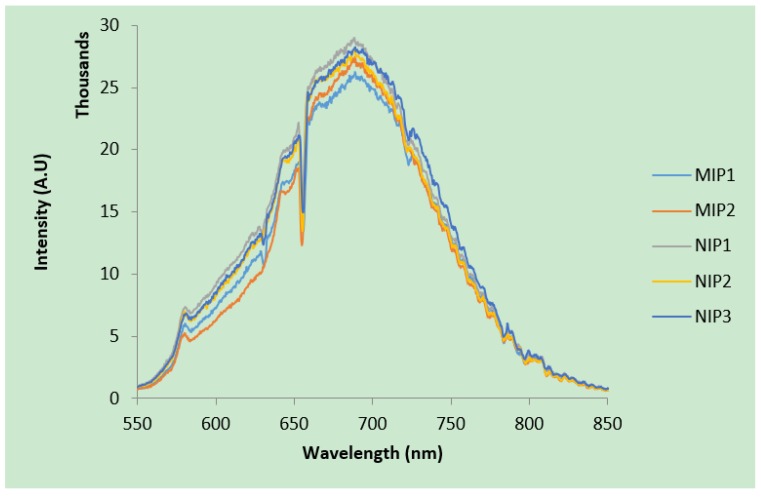
Comparison of emission from similar nanodiamond clusters of 4.8 ± 0.2 μm diameter.

Optical microscopy was used to obtain images of molecularly imprinted polymer and non-imprinted polymer for PAAm hydrogel films. Figure 4a,b shows nanodiamond clusters for both non-imprinted and molecularly imprinted polymers, respectively. The images reveal that nanodiamonds tend to aggregate in clusters. The corresponding particle size and distribution was analyzed using “ImageJ” software (Version 1.47V) with results shown in Figure 4c,d. Both non-imprinted and molecularly imprinted polymers show similar average nanodiamond particle size and distribution (3.4 ± 1.8 µm and 3.1 ± 1.9 µm, respectively). We studied the relation between the cluster size and its fluorescence emission for both molecularly imprinted and non-imprinted polymer hydrogels. We found that the emission intensity of nanodiamond-incorporated hydrogel for a given cluster size is similar to within 2%. The corresponding emission for several nanodiamond clusters of 4.8 ± 0.2 μm diameter is shown in Figure 5. We can estimate the minimum number of nitrogen-vacancy centers per particle based on the manufacturer’s specifications (>300 nitrogen-vacancy centers per 70 nm average particle size). Given this, an average nanodiamond cluster size of 4.8 ± 0.2 μm would yield a minimum of 21,000 ± 860 nitrogen-vacancy centers. However, it should be noted that only those nitrogen-vacancy centers near the nanodiamond surface are likely to impact changes in the surface chemical potential during creatinine binding, thus leading to quenching of the NV^−^/NV^0^ ratio. For this reason, measurements of fluorescence quenching in this study were consistently performed on nanodiamond particles of diameter 4.8 ± 0.2 µm.

### 3.3. Stern-Volmer Plot for Nanodiamond-Incorporated PAAm Hydrogel

Common fluorescence quenching mechanisms include Fluorescence Resonance Energy Transfer (FRET), and collisional or static quenching. In FRET, radiation-less energy transfer can occur from a donor molecule (which absorbs a photon) to an acceptor molecule close to the donor. The probability of energy transfer in FRET is dependent on the overlap between the emission spectrum of the donor and absorption spectrum of the acceptor. FRET efficiency varies with the inverse sixth power of the distance between donor and is therefore a very short-range effect. Since the absorption of creatinine is in the ultraviolet region near 230 nm and our nanodiamond emission is in the range of 560–860 nm, no FRET interaction is expected to occur between nanodiamond and creatinine [20]. However, a chemical potential at the negatively charged nanodiamond surface induced by a positively charged adsorbate such as creatinine can impact fluorescence by changing occupation of nitrogen-vacancy center charge states with interaction range proportional to 1/R^2^ [12]. We investigated how nanodiamonds emission was quenched by first observing how the peak intensity (around 700 nm) changed upon addition of creatinine, and then by observing how the emission intensity from NV^−^ and NV^0^ changed upon creatinine addition.

**Figure 6:**
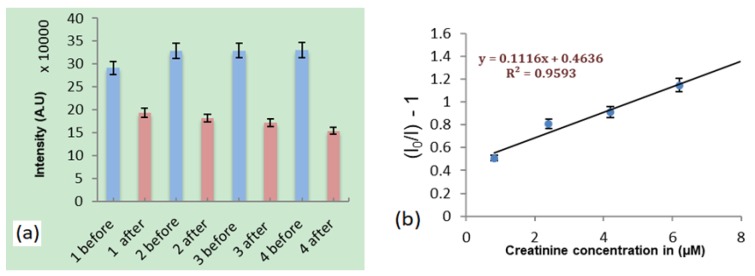
(**a**) Comparison of peak nanodiamond fluorescence intensity (around 700 nm) for molecularly imprinted polymer before and after the addition of four different creatinine concentrations. (**b**) Stern-Volmer plot generated from the data.

Equilibrium adsorption tests were first performed by measuring the nanodiamond fluorescence peak intensity (around 700 nm) from molecularly imprinted polymers for creatinine concentrations of 0.82 µM, 2.40 µM, 4.10 µM and 6.20 µM, separately. In order to minimize uncertainty in fluorescence intensity measurements, the laser spot of 2 µm was consistently focused onto nanodiamond particles of size 4.8 ± 0.2 µm. Results showed nanodiamond fluorescence quenching for all four concentrations as shown in Figure 6a. A Stern-Volmer plot for this data was generated as shown in Figure 6b.

**Figure 7:**
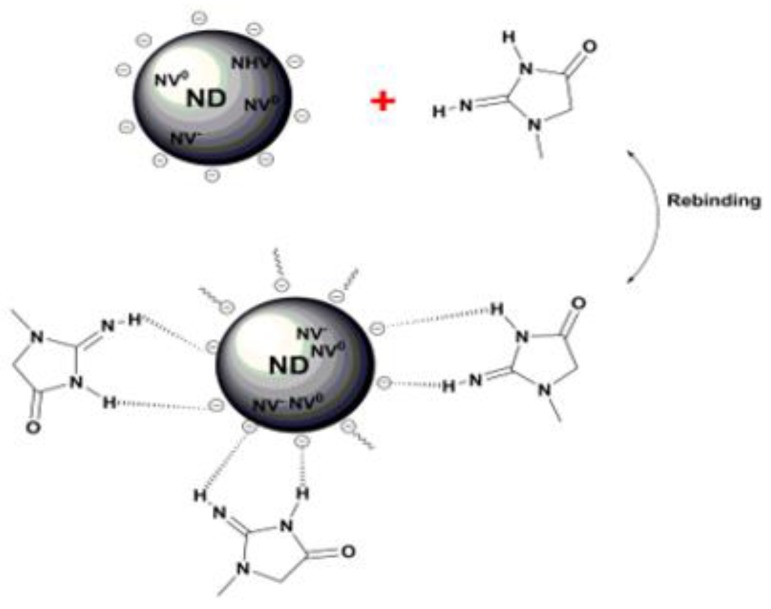
Schematic showing Coulomb interaction between nanodiamonds and creatinine molecules where charge state of near-surface nitrogen-vacancy centers can be affected.

The second way to measure changes in nanodiamond fluorescence from interactions with creatinine in the hydrogel is by detecting emission from the nitrogen-vacancy centers (after subtracting the phonon side band). Nanodiamonds in the molecularly imprinted polymer will undergo changes in surface charge termination after rebinding of creatinine [12,21]. The Coulombic interaction between creatinine molecules (positive) and nanodiamond surface (negative) is represented schematically in Figure 7 and will potentially influence occupation of NV^−^ and NV^0^ states near the nanodiamond surface. In terms of the nanodiamond near-surface band structure, hydrogen termination on undoped diamond surfaces leads to upward band bending (hole accumulation layer) while oxygen termination (with an electric dipole moment in the opposite direction) causes down ward band bending.

**Figure 8:**
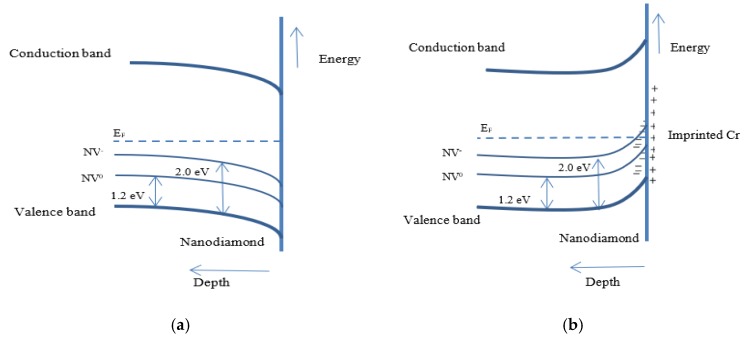
Nanodiamond band structure showing band bending near the surface (**a**) before creatinine addition with negative surface termination resulting in downward band bending and fully occupied nitrogen-vacancy centers and (**b**) after creatinine addition resulting in upward band bending and unoccupied NV^−^ states near the nanodiamond surface.

According to Petráková et al. [12], the ground states of nanodiamond color centers are occupied by a single electron (NV^0^) or two electrons (NV^−^) with 1.2 eV and 2.0 eV ground state energy, respectively, as shown in the near-surface band structure schematic of Figure 8a for negatively terminated nanodiamond. Once creatinine is bound to imprinted sites in the vicinity of nanodiamond, electrons are transferred from the valence band to compensate for the positive charge induced at the diamond surface by creatinine. This contributes to an upwards surface band bending shown in Figure 8b. The upward band bending leaves NV^−^ states unoccupied (above the Fermi level) at a shallow distance below the nanodiamond surface and therefore cannot contribute to luminescence [12,22,23]. In this way, we expect to observe a quenching of NV^−^ luminescence relative to NV^0^ once the creatinine is rebound in the molecularly imprinted polymer.

## 4. Measurements of Nanodiamond Quenching

**Figure 9:**
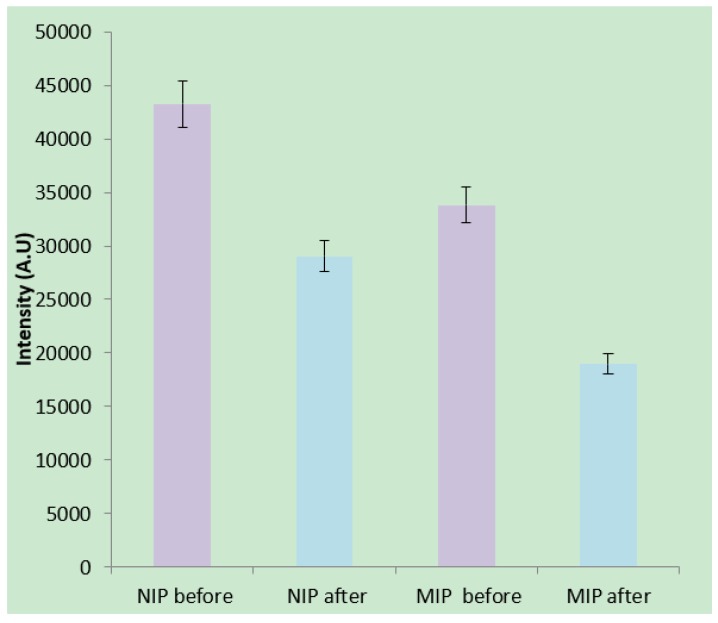
Fluorescence quenching of nanodiamond (peak around 700 nm) for molecularly imprinted and non-imprinted polymers before and after the addition of creatinine.

The imprinting factor is defined as the fluorescence quenching ratio of molecularly imprinted polymer divided by the quenching ratio of non-imprinted polymer after binding with creatinine. Samples of molecularly imprinted and non-imprinted polymer were prepared identically except that no creatinine was initially added for the non-imprinted polymer. The imprinting and rebinding concentration of creatinine was 0.30 mM. Imprinting sites (on the surface or within the hydrogel) for creatinine should therefore only exist inside the molecularly imprinted polymer. We carefully measured the fluorescence of identical sizes of clusters to study the impact that imprinting sites have on quenching of the nanodiamonds. Figure 9 shows measurements of the nanodiamond emission at the peak intensity around 700 nm after exposure to 0.3 mM of creatinine. While both molecularly imprinted and non-imprinted polymers show nanodiamond quenching, the molecularly imprinted polymer is quenched 30% more than the non-imprinted polymer.

Therefore, the calculated imprinting factor was 1.3 ± 0.1. While low, this is comparable to imprinting factors from other studies using fluorescent polymers or quantum dots as a signal transducer for the detection of creatinine [7,24,25]. Not all creatinine molecules can be expected to be totally removed from the hydrogel during washing process as we found previously [16], and this may explain why quenching of the non-imprinted polymer is observed. Nevertheless, the low imprinting factor can likely be improved in several ways including finding ways to optimize washing techniques to remove imprinted creatinine from the hydrogel, to better disperse the nanodiamond particles of nm-size uniformly throughout the hydrogel, and to reduce potential for large pores during hydrogel synthesis which may act to trap non-imprinted creatinine.

**Figure 10:**
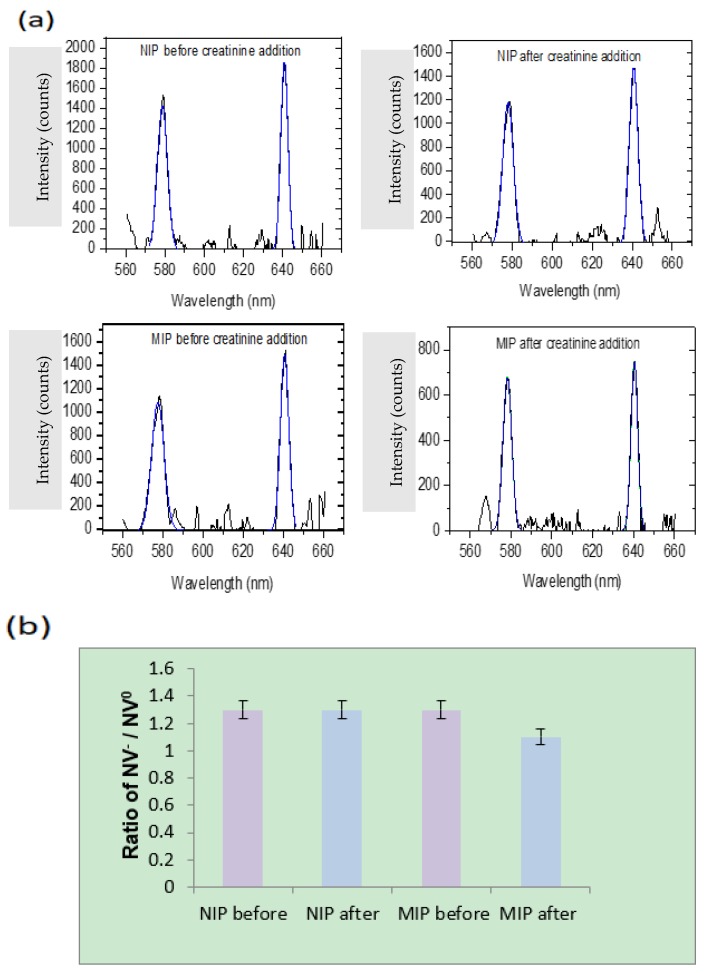
(**a**) Photoluminescence peak fits in the range of nitrogen-vacancy centers after background subtraction and using a Gaussian fitting profile for non-imprinted and molecularly imprinted polymer (before and after creatinine addition). (**b**) Nanodiamond nitrogen-vacancy center intensity ratios (NV^−^/NV^0^) before and after the addition of creatinine for non-imprinted and molecularly imprinted polymers. Error bars indicate standard deviation, n = 9 for both polymers.

As mentioned, the second approach of measuring nanodiamond fluorescence quenching is based on changes in fluorescence from nitrogen-vacancy color centers (NV^−^/NV^0^). Due to the difficulty in resolving the nitrogen-vacancy zero phonon lines from the large phonon side band fluorescence (as shown in Figure 1), we performed a background subtraction of all PL data and fit the zero phonon lines using Gaussian profiles. The resulting peak fits are shown in Figure 10a revealing the significant difference in NV^−^ and NV^0^ peak intensity from before/after addition of creatinine for the molecularly imprinted polymer. As expected, the non-imprinted polymer does not show any appreciable difference. The corresponding NV^−^/NV^0^ ratios are plotted in Figure 10b, with error bars as the standard deviation from n = 9 samples for both molecularly imprinted and non-imprinted polymers. The insignificant change in NV^−^/NV^0^ ratio for the non-imprinted polymer samples indicate that there were no imprinting sites that bring creatinine close enough to interact with nitrogen-vacancy centers at the surface of the nanodiamonds. In contrast, molecularly imprinted polymer samples show a decrease of 15% in the ratio, indicative of a drop in NV^−^ charge occupation as the nanodiamond near-surface band structure bends upward above the Fermi level.

## 5. Conclusions

In this study, we report creatinine detection using an artificial receptor based on a molecularly imprinted polymer method. The sensor was prepared by incorporating fluorescent nanodiamond into a creatinine-imprinted polyacrylamide hydrogel. Quenching of nanodiamond peak fluorescence from the creatinine-imprinted polymer was 30% higher than that of the non-imprinted polymer, yielding an imprinting factor of 1.3 ± 0.1 indicating that the molecularly imprinted polymer has a higher affinity for creatinine than the non-imprinted polymer. Microscopic imaging of the hydrogel showed that nanodiamonds aggregate to form clusters of different diameter. Fourier transform infrared spectroscopy revealed the presence of polyfunctional surface groups such as OH, COOH, ethers, and hydrogen. Creatinine was detected based on nanodiamond-creatinine charge interaction resulting in an expected surface band-bending and quenching of NV^−^ charge states. Results show a significant decrease of NV^−^/NV^0^ emission for the molecularly imprinted polymer while the same emission ratio for the non-imprinted polymer did not change. An advantage of the ratiometric sensor is that it is specific to surface chemical potentials at the near-nanodiamond surface and may be less prone to fluorescence from unwanted background (e.g., polymer-induced fluorescence). The fluorescent sensing is expected to provide reliable sensitivity of trace level quantities of creatinine with a longer range (Coulombic, 1/R^2^) interaction to imprinted sites than that for a sensor based on acceptor/donor resonance energy transfer (FRET, 1/R^6^).

## Figures and Tables

**Table 1:** FTIR peak assignments from as-received nanodiamond.

Transmittance Frequency (cm^−1^)	Assignment
3385	OH stretching, attributed to the hydroxyl groups on nanodiamonds
2940	CH_2_ asymmetric stretching, attributed to the symmetric stretching modes of various CH_x_ groups on nanodiamonds
2841	CH_2_ symmetric stretching, attributed to the asymmetric stretching modes of various CH_x_ groups on nanodiamonds
2335	CO_2_ stretching vibrations
1591	OH bending, attributed to the hydroxyl groups on nanodiamonds
1406	C=O symmetric stretching, attributed to the carboxyl groups on nanodiamonds
1047	C–O stretching, attributed to the hydroxyl and ether groups on nanodiamonds

## References

[1] Mochalin V.N., Shenderova O., Ho D., Gogotsi Y. (2012). The properties and applications of nanodiamonds. Nat. Nanotechnol..

[2] Raty J.-Y., Galli G. (2005). Optical properties and structure of nanodiamonds. J. Electroanal. Chem..

[3] Aleksenskii A., Osipov V.Y., Vul A.Y., Ber B.Y., Smirnov A., Melekhin V., Adriaenssens G., Iakoubovskii K. (2001). Optical properties of nanodiamond layers. Phys. Solid State.

[4] Havlik J., Petrakova V., Rehor I., Petrak V., Gulka M., Stursa J., Kucka J., Ralis J., Rendler T., Lee S.-Y. (2013). Boosting nanodiamond fluorescence: Towards development of brighter probes. Nanoscale.

[5] Yu S.-J., Kang M.-W., Chang H.-C., Chen K.-M., Yu Y.-C. (2005). Bright fluorescent nanodiamonds: No photobleaching and low cytotoxicity. J. Am. Chem. Soc..

[6] Laube C., Oeckinghaus T., Lehnert J., Griebel J., Knolle W., Denisenko A., Kahnt A., Meijer J., Wrachtrup J., Abel B. (2019). Controlling the fluorescence properties of nitrogen vacancy centers in nanodiamonds. Nanoscale.

[7] Ren X., Chen L. (2015). Quantum dots coated with molecularly imprinted polymer as fluorescence probe for detection of cyphenothrin. Biosens. Bioelectron..

[8] Rosenthal S.J., Chang J.C., Kovtun O., McBride J.R., Tomlinson I.D. (2011). Biocompatible quantum dots for biological applications. Chem. Biol..

[9] Kaur R., Badea I. (2013). Nanodiamonds as novel nanomaterials for biomedical applications: Drug delivery and imaging systems. Int. J. Nanomed..

[10] Doherty M.W., Manson N.B., Delaney P., Jelezko F., Wrachtrup J., Hollenberg L.C. (2013). The nitrogen-vacancy colour centre in diamond. Phys. Rep..

[11] Schirhagl R., Chang K., Loretz M., Degen C.L. (2014). Nitrogen-vacancy centers in diamond: Nanoscale sensors for physics and biology. Annu. Rev. Phys. Chem..

[12] Petráková V., Taylor A., Kratochvílová I., Fendrych F., Vacík J., Kučka J., Štursa J., Cígler P., Ledvina M., Fišerová A. (2012). Luminescence of nanodiamond driven by atomic functionalization: Towards novel detection principles. Adv. Funct. Mater..

[13] Killard A.J., Smyth M.R. (2000). Creatinine biosensors: Principles and designs. Trends Biotechnol..

[14] Mohabbati-Kalejahi E., Azimirad V., Bahrami M., Ganbari A. (2012). A review on creatinine measurement techniques. Talanta.

[15] Wang H., Yi J., Velado D., Yu Y., Zhou S. (2015). Immobilization of Carbon Dots in Molecularly Imprinted Microgels for Optical Sensing of Glucose at Physiological pH. ACS Appl. Mater. Interf..

[16] Almotiri R., Catledge S.A. (2019). Molecular Imprinted Polyacrylamide as a Receptor for Creatinine Detection. Adv. Sci. Eng. Med..

[17] Fan Z., Ilnitska H., Lysakovskyi V., Ivakhnenko S., Kovalenko T. (2018). Effect of physicochemical action on the aggregative properties of detonation-synthesized nanodiamonds. IOP Conference Series: Materials Science and Engineering.

[18] Petit T., Puskar L. (2018). FTIR spectroscopy of nanodiamonds: Methods and interpretation. Diam. Relat. Mater..

[19] Patyukova E., Rottreau T., Evans R., Topham P.D., Greenall M.J. (2018). Hydrogen Bonding Aggregation in Acrylamide: Theory and Experiment. Macromolecules.

[20] Tsai H.-A., Syu M.-J. (2005). Synthesis and characterization of creatinine imprinted poly (4-vinylpyridine-co-divinylbenzene) as a specific recognition receptor. Anal. Chim. Acta.

[21] Nebel C.E. (2005). Surface transfer-doping of H-terminated diamond with adsorbates. New Diam. Front Carbon Technol..

[22] Ristein J., Riedel M., Ley L. (2004). Electrochemical surface transfer doping the mechanism behind the surface conductivity of hydrogen-terminated diamond. J. Electrochem. Soc..

[23] Maier F., Riedel M., Mantel B., Ristein J., Ley L. (2000). Origin of surface conductivity in diamond. Phys. Rev. Let..

[24] Syu M.-J., Hsu T.-J., Lin Z.-K. (2010). Synthesis of recognition matrix from 4-methylamino-N-allylnaphthal-imide with fluorescent effect for the imprinting of creatinine. Anal. Chem..

[25] Lin H.-Y., Ho M.-S., Lee M.-H. (2009). Instant formation of molecularly imprinted poly (ethylene-co-vinyl alcohol)/quantum dot composite nanoparticles and their use in one-pot urinalysis. Biosen. Bioelectron..

